# The impact of crisis and COVID-19 on Syrian children growth, health awareness and nutritional practices. a cross-sectional study

**DOI:** 10.1186/s12887-023-04115-w

**Published:** 2023-06-15

**Authors:** Seba Harphoush, Bashar Shahood, Ihab Harphoush, Doris Abra Awudi, Shakeel Ahmad, Margaret Zaitoun, Makhala Mary Weeto, Zhong Li

**Affiliations:** 1 The Key Laboratory of Modern Toxicology of Ministry of Education, School of Public Health, Nanjing Medical University, Nanjing, 211166 China; 2 Department of Nutrition, Faculty of Health Science, Al-Baath University, Homs, Syria; 3 Department of Oral Implantology, Affiliated Hospital of Stomatology, Nanjing Medical University, Nanjing, 210029 PR China; 4 Research Institute of Economic and Management, Southwestern University of Finance and Economic, Chengdu, PR China; 5 Department of Nutrition and Food Hygiene, School of Public Health, Nanjing Medical University, Nanjing, 211166 PR China

**Keywords:** Syrian children, Crisis, Nutrition, Growth, COVID-19 pandemic

## Abstract

**Background:**

Despite the great steadfastness that Syria has achieved in the face of more than a decade of stifling crisis followed by the global COVID-19 pandemic, the heavy impacts of these long rough years are certain and crucial on the health and nutrition levels, specially on vulnerable groups like women and children. Moreover, the lack of research and data on health and nutritional status of children within Syria makes it very difficult to draw conclusions and act effectively. The purpose of the current study was to evaluate growth development and give an insight about the public health awareness and nutritional practices among Syrian primary schools children.

**Methods:**

A cross-sectional study was conducted among private and public primary schools’ students aged 6 to 9 years old in Homs governorate in January to April 2021, anthropometric measures were taken and data assessment of socioeconomic background, nutritional practices, and health awareness was achieved by conducting two surveys answered by parents and students.

**Results:**

We defined the total prevalence of obesity (11.8%), underweight (5.6%) and stunting (13.8%), with a significant increased in underweight and stunting prevalence ,(9%, 21.6%) respectively, among public schools’ students compared to private schools’ students. Differences in nutritional practices and health awareness were recorded between public and private schools’ students under socioeconomic impact.

**Conclusions:**

This study contributes to evaluate the burden of crisis and COVID-19 pandemic on Syrian children growth and health practices in Syria. Improving health awareness and nutritional support among Syrian families to help children meet their growth needs is recommended. Moreover, additional research should be conducted to evaluate micro-nutrients deficiencies and provide appropriate medical support effectively.

**Supplementary Information:**

The online version contains supplementary material available at 10.1186/s12887-023-04115-w.

## Introduction

It’s more than decade since Syria was plunged into a life-threatening conflict, living conditions have deteriorated, and the recent COVID-19 pandemic has exacerbated an already dire situation and placed more burdens on the public health sector. One of the most affected groups are women and children [1], the reality of health and nutritional status of these vulnerable groups is not well recognized and documented [2, 3]. While most of post-crisis literature has been focusing on deteriorated health conditions, nutritional deficiencies, and educational difficulties of Syrian refugees children in neighbouring countries such as Jordan, Lebanon and Turkey, very little has been investigated about the obstacles of children within Syria [4, 5]. Furthermore, the majority of these studies and humanitarian reports inside Syria focused on the conflict areas in Syria with considerable geographic and thematic gaps [6], such as the north of Syria, that areas have suffered the most and are still suffering from the disruption of government services [7]. We do not find any studies in the under-govermental areas where reconstruction and rehabilitation have begun. Moreover, conducting long-term high quality clinical research was almost impossible. University hospitals did not have an electronic medical record system for archiving patients’ data, which makes extracting and verifying patients data difficult and time consuming, and often unreliable, no funding for any kind of research was provided, internet connection was unreliable and slow, and electrical current was unstable with frequent daily interruptions [8].

Prior to the crisis, Syria was on a developing mode, classified as a middle income country, with a robust national health system head by the public sector, offering free medical services around all country's provinces. Syria was on target to meet the Millennium Development Goals in Maternal Mortality Ratio (MMR), Infant Mortality Rate (IMR) , and with a comprehensive vaccination coverage (100% by 2005) [9]. The rapidly growing private sector has made tremendous achievements in the field of health care and the economic situation in the country in general [3]. Furthermore, the education system was effectively progressing with 97% children antecedence in the primary schools, public schools offered free and fine quality education on all over the country.

The heavy years of war have devastated the country’s critical civilian infrastructure and public services, according to WHO recent report 6.7 million people are internally displaced; 5.6 million people have become refugees, mostly in neighboring countries [10]. Economically, the Syrian Pound lost 78 % of its value and food prices increased by 236 % in 2020, leaving up to ninety percent of the population under the poverty line. This is a 10% increase compared to previous years [10, 11]. At least 12.4 million people are in need of health assistance, with the absence of essential health services due infrastructure destruction and low functioning hospitals and health centers [10]. Furthermore, western sanctions and economic embargoes had massively hampered health services [12, 13]. Half a million children are chronically malnourished and an additional 137000 children under five years of age are suffering from acute malnutrition, heightening their exposure to preventable morbidity and mortality. Child labour and child marriage reported as frequently occurring in 22% and 18% of assessed communities, respectively [10, 14]. Only one third of schools are fully functional, and with schooling becoming unaffordable, 2.45 Million children were out of school in 2020 [11, 14]. Voices have been rising calling international forces to act and save children lives in Syria and end sanctions and violence [15].

The attention to the role of nutrition was in its infancy in Syria prior to the crisis. The Health Sciences collage, which includes the Department of Nutrition, first of its kind in Syria, was established in 2005 by virtue of Republican Decree No. 274 dated 30/1/2005 at AL-Baath University. Moreover, nutrition was not a priority before the crisis considering the low prevalence of global acute malnutrition (GAM) under 5 years old, and the very limited information on nutrition situation, nutritional assessment tools, and feeding practices especially for infants and young children [16]. The nutrition situation evaluation was very limited prior to the crisis, very few reports are available on Syrian children nutritional status and nutrients sufficiency. In the purpose of increasing the country's nutritional profile, the nutrition Sector was established in Syria (Damascus) in 2013 with the cooperation of few partners; these were mainly UN agencies (UNICEF, World Food Programme (WFP) and WHO), the Ministry of Health (MoH) and the Syrian Arab Red Crescent (SARC) [16]. In 2015-2016 the Nutrition Sector, through the MoH and with the financial and technical support of UNICEF, conducted SMART nutrition surveys in accessible areas, including 11 of the 14 governorates in Syria. The SMART surveys identified an acceptable level of global acute malnutrition (GAM) of 3% and severe acute malnutrition (SAM) of 0.6 % in children, moderate levels of acute malnutrition among women (7.8 percent), moderate public health problem levels of anaemia among both women and children, and poor infant and young child feeding practices [17]. In 2019 a nutrition SMART survey was conducted, showing moderate levels of GAM (0.4 %) and stunting (12 %), while maternal anemia (27%) and micro-nutrients deficiencies in children showed high values [18].

It's very expected that COVID-19 pandemic, which has exposed tremendous disability in epidemic response and highlighted the fragility of public health systems throughout the world, will have a devastating impact on under-war countries such as Syria where these weaknesses are more apparent [19]. In Syria, where conflict has been succeeded by sanctions and other, more coercive measures [13], widespread cooperation to ensure efficient delivery of medicines and equipment to combat COVID-19 in Syria is lacking. Number of infected cases is very hard to be detected as the country has very limited resources to conduct tests on a large scale, the demolished public health system is lacking equipment, human resources, and necessary preparedness to contain increasing confirmed cases [20]. Moreover, the majority of Syrian population live in poverty, such deteriorated human conditions will reflect on the ability to follow hygiene and prevention measurements, especially in northern areas where people displaced internally and are suffering from overcrowding, poor hygiene, such poor livelihoods will facilitate the virus spread [20]. This pandemic has increased stress and life hardness for Syrians inside Syria [21, 22]. One truth that has been proved that COVID-19 still down the list of life problems and fears to face for Syrians.

## Methods

### Design and sampling method

This cross sectional study used primary data sources, and was conducted at Homs city, which is a city in western Syria and the capital of the Homs Governorate. It is located 162 kilometres north of Damascus, on the Orontes River, Homs is also the central link between the interior cities and the Mediterranean coast. The city has been severely affected by the conflict in Syria.

Most of the recent studies that discuss the nutritional status of Syrian children have taken place within the displaced groups outside the Syrian borders or within areas that have been severely affected by the war, such as northern Syria, we do not find many studies on the health of children in safe areas where government services are still in effect. Homs city is an average example of life in Syria, where the city was affected by 50% of the war in terms of the destruction of the infrastructure, but reconstruction has begun and the city witnessed the return of the population at a high rate recently. In addition to the average economic income of the Syrian family in this city, where the rich live next to the poor, majority of children in this city have returned to school, whether in public or private schools, with a relative increase in child labor and the phenomenon of beggary. Our goal was to give an insight about the public health awareness and nutritional practices between Syrian primary schools' children, and evaluate the associated risk factors.

According to the directorate of education at Homs governorate, department of Statistics, the total number of fully functioning primary schools in 2000 was 151 public schools and 10 private schools, in 2010 pre-crisis the total number of fully functioning primary schools was 207 public schools and 24 private schools, in 2021 post-crisis this number has decreased to 138 public schools and 9 private schools. It’s estimated that 2.45M Syrian children were out of school in 2020 [14].

Although rehabilitation has begun and back-to-school campaign is in full swing, the remnants of the crisis on education and educational institutions are still huge and need a lot of financial and human support on the ground.

For the purpose of this study, several private and public primary schools were selected randomly. The data for surveys and anthropometric measurements were collected directly from a sample of Syrian schools’ students registered on first, second, and third grade in the chosen schools. Based on the number of total registered students on first, second and third grades on the Directorate of Education in Homs governorate, which is 23330 students, a required sample of 378 Students was estimated at a 95% confidence interval (95% CI) with a 5% margin of error. The sample size was adjusted with an estimated design effect of 1.5 for the sampling design (*N* = 576). Considering an estimated non-response rate of 15%, a total of 662 students were targeted for the survey filled by students’ parents (Table S1). Another survey was designed to be answered by only third grade students ( Table S2), as it required a certain ability to answer. All questions were reviewed by two health specialists and 3 schools’ teachers from the participating schools and were also pre-tested. The present study was conducted through January 2021 after receiving approval from schools administrations and Directorate of Education in Homs governorate.

### Anthropometric measurements

Height, weight, and age for students registered in our study were taken by trained research assistants. Weight was measured to the nearest 0.1 kg in light indoor clothing and with bare feet or stockings, using a portable standard calibrated balance and height was measured, without shoes, to the nearest 0.1 cm using a portable stadiometer. Body mass index (BMI) (kg/m2) was calculated by dividing the weight (kg) over the height squared (m2). Age and gender-specific BMI z-scores (BAZ), height for age z-scores (HAZ), and weight for age z-scores (WAZ) were calculated for children using the WHO Anthro Plus software (1.0.4) according to WHO Child Growth Standards. Children were classified as thinned, normal, overweight, obese, and stunted, based on the WHO age and gender-specific cut-offs [23], Stunting and underweight were defined as HAZ < –2 SD and WAZ < –2 SD, respectively. Obesity and overweight were defined as BAZ > +2 and +1 < BAZ ≤ +2, respectively. Thinness was defined as BAZ < -2.

### Surveys design

Parents survey was designed to collect information in several aspects, in order to evaluate and describe the families socioeconomic situation, educational health background, children nutritional status, and dietary habits. In addition, parents’ nutritional knowledge and health awareness were evaluated by true and false statements, and a knowledge score out of 10 was calculated. In the same manner, children’ health knowledge and attitude were evaluated through a survey answered by third grade students, knowledge score out of 10 and attitude score out of 8 were calculated, in addition to dietary habits and life style.

### Statistical analysis

Data analysis was performed using the Statistical Package for Social Sciences, version 27.0 (SPSS Inc., Chicago, IL, USA). Descriptive statistics were performed for continuous and categorical variables. The number of subjects and percentages (n, %) represented nominal variables, whereas mean and standard deviation (mean ± SD) were used to represent continuous variables. Chi-square test was used to analyze group differences for categorical variables and one-way ANOVA test to compare means. Statistical significant was defined as *p*-value < 0.05.

## Results

There were 526 valid answered surveys by parents of 662 distributed surveys targeting students’ parents (50.2% female students, 49.8% male students) divided to (320 from private schools, 206 from public schools), 224 valid answered surveys by third grade students (124 private, 100 public), 340 fully recorded anthropometric measurements (206 private, 134 public) (Table 1). This study was conducted at the beginning of 2021, when COVID-19 epidemic was at its peak in Syria, the percentage of student attendance in primary public schools was very low, as measures to reduce Corona virus spread in public schools were almost non-existent, while private schools encouraged more attendance by providing some relatively preventive measures.

**Table 1:** Anthropometric measurements

	Total		school	Mean	Std. Deviation	*p*-value
	Mean	Std. Deviation				
Age (years)	6.97	0.83	Private	6.94	0.82	0.215
			Public	7.03	0.85	
Weight (Kg)	25.73	7.59	Private	25.97	8.59	0.40
			Public	25.32	5.5	
Height (cm)	123.17	11.84	Private	123.49	11.40	0.53
			Public	122.69	12.51	
BMI (kg/m^2^)	16.67	2.83	Private	16.77	3.59	0.65
			Public	17.35	4.04	

*N*= 340 (206 private, 134 public), (172 female,168 male)

### Anthropometric assessment results

As shown in Table 2, WAZ and HAZ were significantly increased for private schools’ students compared to public schools’ students ( *P* < 0.01, *P* < 0.001) respectively, while BAZ had no significant different between private and public schools’ students. WAZ, HAZ, and BAZ had no significant difference based on gender at our study age range (6-9 years old)

**Table 2:** Anthropometric measurements based on Z-score system

	School	Mean	Std. Deviation	*P* value	Gender	Mean	Std. Deviation	*P* value
WAZ	private	0.0422	1.215	0.007	female	-0.214	1.321	0.087
	puplic	-0.325	1.208		male	0.013	1.256	
Total		-0.102	1.224					
HAZ	private	-0.289	1.406	0.000	female	-0.593	1.321	0.212
	puplic	-0.823	1.308		male	-0.404	1.458	
Total		-0.5	1.391					
BAZ	private	0.234	1.478	0.9	female	0.128	1.535	0.213
	puplic	0.213	1.494		male	0.328	1.423	
Total		0.226	1.482					

*N*= 340 (206 private, 134 public), (172 female,168 male)

As shown in Table 3, Children growth status was classified based on BAZ to normal, overweight, obesity, and thinness. The distribution of these categories showed no significant difference between private and public schools. On the other hand, the stunting prevalence was significantly higher (*p* = 0.001) for public schools 21.6% compared to private schools 8.7 %. Moreover, underweight was significantly increased (*p* = 0.029) for public schools compared to private schools (9%, 3.4 %), respectively. There were no significant gender differences of these categories’ distribution.

**Table 3:** Growth classifications based on anthropometric assessment

School	Count	Normal	Obesity	Overweight	Thinness	*p*-value	Underweight	*p*-value	Normal	Stunted	*p*-value
Private	n	123	25	42	16	0.908	7	0.029	188	18	0.001
	%	59.7	12.1	20.4	7.8		3.4		91.3	8.7	
Public	n	77	15	29	13		12		105	29	
	%	57.5	11.2	21.6	9.7		9		78.4	21.6	
Total	n	200	40	71	11		19		293	47	
	%	58.8	11.8	20.9	3.2		5.6		86.2	13.8	

*N*= 340 (206 private, 134 public), (172 female,168 male)

### Parents survey results

After analyzing parents responds, we found the following socioeconomic results, as shown in Table 4. The current financial situation was significantly better in private schools compared to public schools, and COVID-19 pandemic crisis significantly caused more damage on parents’ financial situation within public schools than parent within private school. Moreover, educational level was significantly different between public and private schools’ parents, noticeably we found higher rate of post-graduation studies among private schools’ parents compared to public schools’ parents. In compatible with that, There was a strong significant difference between knowledge score (*p* < 0.0001) between private and public schools’ parents (7.76 ± 0.1, 6.79 ± 0.14) respectively. According to our results, 50.3% of mothers breastfed for one year or more and 68.14% breastfed for at least six months. Parents smoking rate was 61% (55.6% for men, 18.9% for women), and smoking significantly increased in private schools compared to public schools. Mean sleeping hours was 8.55 ± 0.04 hours with a significant difference (*p* = 0.021) between private and public schools.

**Table 4:** Socioeconomic assessment

	Mean	school	N	Mean	Std. Deviation		*p*-value.
Financial situation On scale from 1 To 5	3.27	Private	314	3.61	0.82		0.000
		Public	206	2.74	1.02		
COVID-19 negative impact on financial situation On scale from 1 To 5	2.98	Private	314	2.72	1.37		0.000
		Public	205	3.37	1.46		
Knowledge Score	7.3784	Private	313	7.76	1.78		0.000
		Public	205	6.8	2.05		
Sleeping hours	8.55	Private	318	8.61	0.798		0.032
		Public	205	8.45	0.795		
				school		Total	*p*-value.
				Private	Public		
Mother's educational level		No education	n	4	4	8	0.038
			%	1.2	1.9	1.5	
		High school	n	121	70	191	
			%	37.8	34.0	36.3	
		Bachelor	n	162	124	286	
			%	50.6	60.2	54.4	
		Master/PhD	n	30	8	38	
			%	9.4	3.9	7.2	
Total			N	217	206	523	
Father's educational level		No education	n	7	2	9	0.015
			%	2.2	1.0	1.7	
		High school	n	116	93	209	
			%	36.2	45.1	39.7	
		Bachelor	n	160	103	263	
			%	50.0	50.0	50.0	
		Master/PhD	n	34	7	41	
			%	10.6	3.4	7.8	
Total			N	317	205	522	
Parents’ smoking status		Both parents are not smokers	n	124	91	215	0.023
			%	39.0	44.2	41.0	
		Only mother is a smoker	n	15	3	18	
			%	4.7	1.5	3.4	
		Only father is a smoker	n	121	89	210	
			%	38.1	43.2	40.1	
		Both parents are smokers	n	58	23	81	
			%	18.2	11.2	15.5	
Total			N	318	206	524	
Breastfeeding period		No Breastfeeding at all	n	44	22	66	0.598
			%	13.9	10.7	12.7	
		Less than 6 months	n	61	39	100	
			%	19.3	19.0	19.2	
		More than 6 months	n	52	41	93	
			%	16.5	20.0	17.9	
		A year or more	n	159	103	262	
			%	50.3	50.2	50.3	
Total			N	316	205	521	

knowledge score was calculated based on 10 (tick or cross) statements Table S1 (parents questionnaire module)

Supplements’ usage was evaluated in parents survey (Table 5), consuming micro-nutrients supplements was significantly increased within private schools compared to public schools. Parents in public schools reported that their children aren’t having enough nutrients in their diet by 70.9%. Despite 58.8 % of families were not consuming micro-nutrients supplements at time of conducting this study, 53.3 % of families increased their intake of supplements after COVID-19 spread. First cause of taking micro-nutrients supplements was to increase immunity (70.9% with private schools, 49.3% with public schools), and first cause of not taking supplements was preferring natural sources (79.3% with private schools, 55.6% with public schools), noticeably 39.9% of public schools’ parents did not use supplements for their children because of its expensive cost.

**Table 5:** Micro-nutrients supplements usage

			School				Total	*p*-value.
					Private	Public		
Do you give your child any micro-nutrients supplements?			No	n	171	134	305	0.023
				%	54.6	65.0	58.8	
			Yes	n	142	72	214	
				%	45.4	35.0	41.2	
Total				N	313	206	519	0.000
Do you think your child gets enough vitamins through his/her food?			No	n	139	146	285	
				%	43.6	70.9	54.3	
			Yes	n	180	60	240	
				%	56.4	29.1	45.7	
Total				N	319	206	525	
Has your family's usage of micro-nutrients supplements increased after the spread of Corona in Syria?			No	n	132	112	244	0.006
				%	41.6	54.4	46.7	
			Yes	n	185	94	279	
				%	58.4	45.6	53.3	
Total				N	317	206	523	
If you give your child supplements, what is the reason?								
			Reasons of taking supplements				Total	
			To increase immunity	prescription	To improve mental ability	To make up for the deficiency		
school	Private	n	100	10	21	27	141	
		%	70.9	7.1	14.9	19.1		
	Public	n	36	6	22	17	73	
		%	49.3	8.2	30.1	23.3		
Total		N	136	16	43	44	214	
		%	63.6	7.5	20.1	20.6	100.0	
			Reasons of not taking supplements				Total	
			No need	I don't think it is good for health	Very expensive	I prefer Natural sources of vitamins		
school	Private	n	31	6	11	134	169	
		%	18.3	3.6	6.5	79.3		
	Public	n	10	4	53	75	135	
		%	7.4	3.0	39.3	55.6		
Total		N	41	10	64	209	304	
		%	13.5	3.3	21.1	68.8	100.0	

Percentages and totals are based on respondents. Dichotomy group tabulated at value 1.

In our study we investigated the prevalence of some common children diseases and malnutrition symptoms (Table 6), we found increased levels of anemia and dental caries among public schools’ students compared to private schools’ students, total diagnosed anemia prevalence was 2.8% (1.6% private, 4.9% public). Most common symptoms of micro-nutrients’ deficiencies were frequent constipation and angular cheilitis. In general most symptoms were increased within public schools compared to private schools, except for hair loss which was increased for private schools’ students. the prevalence of dental caries was about 26.5% with noticeably higher prevalence at public schools 37.4% compared to 21% at private schools. Chronic respiratory problems were third on the list with same prevalence in both public and private schools, while frequent infections were increased at public schools compared to private schools. In term of behavioral signs of stress, in both public and private schools we found relatively high prevalence of anorexia (11.7%), nervousness or violence (10.7%), difficulties in academic achievement (9.8%), and tense or concentration difficulties (9.2%).

**Table 6:** Children health assessment

Does your child currently suffer from one of the following diseases?										Total
			Chronic respiratory problems	Diabetes	Anemia	Rickets or growth problems	Frequent infections	Dental problems	Nothing from the list	
school	Private	n	11	0	5	2	3	67	238	319
		%	3.4	0	1.6	.6	.9	21.0	74.6	
	Public	n	7	0	10	2	4	77	118	206
		%	3.4	0	4.9	1.0	1.9	37.4	57.3	
Total		N	18	0	15	4	7	144	356	525
		%	3.3	0	2.8	.7	1.3	26.5	65.4	100.0
Do you notice one of these behavioral signs on your child?										Total
			(Anorexia) Lack of appetite	Insomnia or Sleep Difficulties	Fatigue and Weakness	Tense or concentrate Difficulties	Nervousness or Violence	Difficulties in academic achievement	Nothing from the list	
school	Private	n	33	2	1	29	37	33	219	318
		%	10.4	.6	.3	9.1	11.6	10.4	68.9	
	Public	n	28	3	6	19	19	18	133	204
		%	13.7	1.5	2.9	9.3	9.3	8.8	65.2	
Total		n	61	5	7	48	56	51	352	522
		%	11.7	1.0	1.3	9.2	10.7	9.8	67.4	100.0
Have you noticed the following symptoms on your child?										
			Frequent constipation	Frequent bruises	Hair loss	Muscle pain	Bow legs	Angular cheilitis	Nothing from this list	Total
school	Private	n	7	2	9	4	0	4	294	318
		%	2.2	.6	2.8	1.3	0	1.3	92.5	
	public	n	7	4	1	6	1	10	180	205
		%	3.4	2.0	.5	2.9	.5	4.9	87.8	
Total		N	14	6	10	10	1	14	474	523
		%	2.7	1.1	1.9	1.9	0.2	2.7	90.6	100.0

Percentages and totals are based on respondents. Dichotomy group tabulated at value 1

We assessed the children’ dietary intake through parents’ responds and children’ responds. In parents’ responds, we found significant differences between public and private schools’ students (Table 7), the intake of some basic food needs like fruit, meat sources, and iodized salt was significantly increased for private schools compared to public schools, in addition to junk food intake, while escaping breakfast was significantly increased for public schools compared to private schools.

**Table 7:** Healthy and dietary habits assessment (parents survey)

	N		Mean	Std. Deviation	school	n	Mean	Std. Deviation	*p*-value.
How often does your child eat fruits?	526	Min 0	2.89	0.981	Private	320	3.15	0.76	0.000
		Max 4			Public	206	2.49	1.14	
How often does your child drink milk?	526	Min 0	2.30	1.08	Private	320	2.52	1.04	0.746
		Max 4			Public	206	1.95	1.06	
How often does your child eat fish or other seafood?	526	Min 0	1.52	1.08	Private	320	1.80	1.08	0.000
		Max 4			Public	206	1.09	0.94	
How often does your child eat read meat ?	525	Min 0	2.28	1.19	Private	319	2.70	1.05	0.025
		Max 4			Public	206	1.63	1.09	
Do you use iodized salt in food preparation?	526	Min 0	2.58	1.46	Private	320	2.83	1.34	0.000
		Max 4			Public	206	2.19	1.56	
How often does your child eat breakfast before school?	526	Min 0	2.24	1.56	Private	320	2.48	1.55	0.000
		Max 4			Public	206	1.87	1.49	
How often does your child eat unhealthy foods such as Soda, Candy, and chips…?	524	Min 0	1.68	1.12	Private	318	1.85	1.08	0.000
		Max 4			Public	206	1.43	1.11	

### Students survey results

As shown on Table 8, the results indicated higher score of healthy attitude (*p* = 0.047) and higher score of health knowledge (*p* = 0.001) for private schools’ students compared to public schools’ students. In term of some foods intake, we found no differences in milk and dairy products consumption. Vegetables consumption had also no difference but fruit consumption was significantly increased for private schools compared to public schools in compatible with parents responds. In field of physical activities, frequency of practicing sports was significantly increased for public schools compared to private schools, while there was no difference in frequency of watching TV.

**Table 8:** Healthy and dietary habits assessment (students survey)

	N		Mean	Std. Deviation	school	n	Mean	Std. Deviation	*p*-value.
Attitude Score	224	Out of 8	7.54	0.79	Private	124	7.64	0.69	0.047
					Public	100	7.42	0.89	
Knowledge Score	224	Out of 10	7.82	1.49	Private	124	8.13	1.32	0.001
					Public	100	7.45	1.60	
How often do you eat vegetables?	223	Min 0	3.07	0.83	Private	123	3.15	0.83	0.130
		Max 4			Public	100	2.97	0.82	
How often do you eat fruit?	221	Min 0	3.00	0.91	Private	121	3.12	0.92	0.026
		Max 4			Public	100	2.86	0.88	
How often do you eat dairy foods?	222	Min 0	2.79	1.06	Private	123	2.80	1.05	0.949
		Max 4			Public	99	2.78	1.07	
How often do you watch TV?	222	Min 0	1.72	0.99	Private	122	1.69	0.95	0.667
		Max 3			Public	100	1.76	1.04	
How often do you practice any kind of sports?	222	Min 0	3.31	0.99	Private	122	3.1967	1.00115	0.011
		Max 4			Public	100	3.4500	.97830	

Child Attitude Score and Knowledge Score were calculated based on groups of questions answered by the third grade students (Table S2)

## Discussion

Reviewing literature, there is a huge defect in children growth assessment using anthropomorphic indices and nutritional status evaluation within Syria, and no study was conducted for primary school age children. According to the UNICEF the pre-crisis prevalence of stunting , wasting, and underweight were estimated to be 23% , 9.3%, and 10.3% respectively [24]. According to a recent UNOCHA humanitarian report the post-crisis national prevalence of stunting in children was estimated to be at 12.6 percent, with emergency thresholds reached the in sub-districts of Deir-Ez-Zor and Al-Hasakeh (one in five children stunted), Idleb and Aleppo (one in six children stunted) [25]. In northwest Syria camps, stunting was prevalent in more than 25% of children [26]. According to our assessment, the prevalence of stunting was estimated to be 13.8% for primary school age children in Homs city, with a significantly higher prevalence for public schools 21.6% and significantly decreased HAZ compared to private schools, in the same manner the prevalence of underweight was higher in public schools with significantly decreased WAZ compared to private schools. Those results imply to a chronic malnutrition based on socioeconomic indicators. According to human organizations, the number of food insecure people in Syria has increased by 22 percent, from 2019 to 2020, since mid-2019 impotence to meet basic needs reached 82% of families. Almost 600,000 children were chronically malnourished and 90,000 children were acutely malnourished [14]. No wonder that COVID-19 pandemic, that impaired food security globally, will put most pressure on poor economic communities where risk of deteriorated nutritional status will grow among vulnerable groups like women and children [27]. It was estimated that 300,000 jobs lost since the beginning of the COVID-19 pandemic in Syria [14]. Pandemic situation confrontation depends basically on community preventive measurements, social awareness, and individuals immunity system. Such essentials became exorbitant in the Syrian community, which is panting to recover from the destructive, economic and psychological effects of war. Our results were compatible with a previous study that indicated positive and significant correlations between knowledge, attitudes, and practices among Syrians inside Syria in the field of COVID-19 pandemic [28]. This study is an indication of the uneven living situations in Syrian society and refers to a socioeconomic impact on food availability and dietary patterns, which reflects on the health and nutritional status of children. The gap between rich and poor people is growing with increasing number of Syrians unable to meet their daily needs and provide their children appropriate diet to grow well.

Furthermore, the recent crisis grew more obstacles for Syrian mothers and their infants, low breastfeeding rate in our results was compatible with a previous cross sectional study conducted in Syria post-crisis that found 68.6% of mothers were exclusive breastfeeding at 4 months but this ratio dropped to 12.9% at six months [29]. Moreover, the percentage of smoking still high in the Syrian society, a recent study reported the prevalence of smoking among Syrians to be 58.3 % (61.7 % males vs 38.3 % females) [30]. The association between parental smoking and respiratory symptoms in children was previously recorded in Syria [31]. Although, there was a higher rate of smoking fathers in public schools but the total rate of smoking parents in private school was significantly higher compared to public schools, and noticeably smoking mothers rate was higher in private schools compared to public schools which indicate that financial situation has a huge impact on smoking choice in Syria.

Sleep disorders are very common effects of wars and violence traumas and Syrians had lived one of the cruelest violence [32]. Our study is an evidence that children in Syria and especially in public schools are facing a lot of stress and inferior living atmosphere which means more attention should be paid to the psycho-social effects of this crisis on children mental health and investigations are urgent in this aspect. Malnutrition is a part of a vicious cycle that also includes poverty and disease, the three components being interrelated and each contributing to the occurrence and persistence of the others. Recently, studies indicated rising in multi health disorders in Syrian population, with war related under-laying factors and massive burden on vulnerable groups such as women and children [14, 33]. Vaccines related diseases and infections have spread between internal displaced Syrian children and refugees children especially in campuses [34]. Post-crisis reports indicated rise in anemia prevalence between under under five years old children, pregnant and lactating women [14]. High levels of anemia were documented among Syrian children in different ages within and out of Syria [35, 36]. A recent cross-sectional epidemiological survey in Damascus city [37], indicated that caries prevalence to be (79.1%) among 8 to 12 years old schoolchildren based on clinical examination, and in compatible with our study the reasons of this high levels of caries between children especially in public school were post-crisis growing malnutrition, deteriorated living conditions, negative nutritional practices, and poor health services. Children dental caries is developing rapidly post crisis in Syria [38], a characteristic of the epidemiological transition, community based emergency public health interventions should be taken effectively. Reports indicated that Infections were the largest contributor to children morbidity [7]. Respiratory infections have been documented in Syria through conflict mostly in war zones [39]. Moreover, Syrian children mental and emotional health is one of the biggest concerns after this long destructive crisis, child rights violations have been reported overall Syria, indicating critical threat for children mental and physical health. According to human organizations 27% of households reported signs of distress between their children [14]. High prevalence of mental and emotional health disorders has been recorded among children in north Syria related to displacement and poverty [40]. Moreover, It’s very expected that micro-nutrients deficiencies will reach its upper limits on the health of women and children post crisis. Inadequate food intake and disease are the direct causes of malnutrition, while poverty which connected to low parental education, with deteriorated living conditions are the distal factors. Moreover, culture factors influenced by social and economic background played an important role in the etiology of poor growth like low breastfeeding rate and duration, parental smoking, poor infant and young child feeding practices, lack of sanitation and good hygiene practices, and impaired dietary habits and life style.

### Limitations of the study

The current study has several limitations which provides opportunities for future research. First, the data was collected based on surveys filled by parents and children, face to face interviews will help collect more valuable and true information about nutrition intake and health conditions. Secondly, this study lacks to biochemical Measurements which is necessary to provide a real picture on the nutrients deficiencies levels in order to provide valuable actions to help those children meet their nutrition needs and growth goals. Thirdly, our study was conducted in one governorate, data from all over Syria must be collected to reflect the national situation of the country and represent differences between governorates that have been affected in varying degrees by the crisis.

## Conclusion

Despite numerous limitations, this is the first study to be conducted within Syria in purpose of growth, health and nutritional practices assessment for Syrian primary schools’ children, and first study to compare between private and public schools’ students in Syria. Our results define a critical level of stunting among public primary schools’ students which refer to insufficient dietary intake. The hazards of food insecurity and nutrients deficiencies on children within Syria are still rising under the collapsed economic conditions. By comparing public and private schools' students, we revealed growing recede in the living conditions and food security in the society, which left a large segment of children vulnerable to stunted growth and impaired physical and mental development. The huge impact of Syrian crisis followed by COVID-19 epidemic on Syrians living reality within Syria is hard to describe or measure, a lot of work must be done on the level of data collection. Actions must be taken effectively to rise health conscious between parents and family members. This whole generation born and grew up under crisis lesion needs to join hands and make a great efforts to rehabilitate on the educational, health, and living levels.

It is necessary to provide facilities with financial and technical support for community based health research to provide a better picture of the health reality, which helps in determining the most effective measures for intervention and improve the nutritional status of Syrian children.

Schools constitute an important platform for providing health and nutritional support to children and their families. It is important and useful to have a growth monitoring department under the supervision of a nutritionist and health specialists to monitor the growth of children periodically, which provides data that helps in conducting necessary community based studies and facilitates individual assistance when necessary.

## Supplementary Information

**Additional file 1: Table S1.** Parents questionnaire module.

**Additional file 2: Table S2.** Child questionnaire module.

## Data Availability

Data available on request from the authors: The data that support the findings of this study are available from the corresponding author upon reasonable request.
