# Supplementary material for: The impact of crisis and COVID-19 on Syrian children growth, health awareness and nutritional practices. a cross-sectional study
Source: BMC Pediatr. 2023 Jun 15;23:295. doi: 10.1186/s12887-023-04115-w (PMC10268436; doi:10.1186/s12887-023-04115-w)
Supplement: Supplementary file 1 — Additional file 1: Table S1. Parents questionnaire module. [file 12887_2023_4115_MOESM1_ESM.pdf]

Table S1: Parents questionnaire module

Serial number:	School :	Grade:
Child Name:	Child Gender:	Child Birth date:
Child Weight:	Child Height:	

Please fill the following questions by parents or child direct care provider

What is the mother's educational level?

No education	High school	bachelor	Master or PhD

What is the father's educational level?

No education	High school	bachelor	Master or PhD

How do you describe your financial situation?

Very bad	Not bad	fair	good	Very good

On a scale from 1 to 5, how much has your financial situation affected by Corona disease?

Never affected 1 2 3 4 5 very affected

Do you give your child any supportive vitamins? If your answer is yes, what is the reason?

Yes, a prescription	To increase immunity	Yes, to improve mental ability	Yes, to make up for the deficiency

If you do not give your child any vitamins, why?

No need	I don't think it is good for health	Very expensive	i prefer Natural sources of vitamins

Do you think your child gets enough vitamins through his/her food?

Yes	No	Not sure

Has your family's use of vitamins increased after the spread of Corona disease in Syria?

Yes	No

Does your child currently suffer from one of the following diseases?

Chronic respiratory problems	Diabetes	Anemia	Rickets or growth problems
Frequent infections	Dental problems (delayed eruption or multiple caries)		Nothing from this list

Do you notice one of these behavioral signs on your child?

Lack of appetite	Insomnia or Sleep Difficulties	Fatigue and weakness	Tense or difficulty concentrating
Nervousness or violence	Difficulties in academic achievement		Nothing from this list

Have you noticed the following symptoms on your child? Several answers can be selected

Frequent constipation	Frequent bruises	Hair loss	Muscle pain
Bow legs	Cracks or inflammation at the corners of the mouth (Angular cheilitis)		Nothing from this list

How long has breastfeeding been used for your baby as an infant?

No breastfeeding	Less than 6 months	More than 6 months	A year or more

How many hours does your child sleep?

Less than 6 hours	6-7 hours	7-8 hours	9-10 hours	11-12 hours

does one or both of the parents smoke?

Both parents are smokers	Only father is a smoker	Only mother is a smoker	Both parents are not smokers

Healthy and Dietary habits assessment:

Does your child drink milk?

never	rarely	sometimes	often	always

Does your child eat fish or other seafood?

never	rarely	sometimes	often	always

Does your child eat red meat or liver?

never	rarely	sometimes	often	always

Do you use iodized salt in food preparation?

never	rarely	sometimes	often	always

Does your child eat breakfast before school?

never	rarely	sometimes	often	always

How often does your child eat fruits?

never	rarely	sometimes	often	always

Does your child eat unhealthy foods such as Soda, Candy, and chips…?

never	rarely	sometimes	often	always

Answer the following statements, with true or false (Knowledge score)

The main source of Vitamin D is seafood	√	×
Citrus fruits enhance dietary iron absorption	√	×
Vitamin D deficiency can be associated with obesity	√	×
Excessive drinking of milk leads to child iron deficiency anemia	√	×
Egg yolks are rich in iron and vitamin D.	√	×
Rickets is a disease caused by a lack of vitamin E	√	×
Breast milk is completely sufficient for the baby during the first six months without any additional food	√	×
Liver is an excellent source of vitamin B12	√	×
The main source of vitamin A is sun exposure	√	×
Vitamin K is critical for vision function	√	×

correct answer was counted as 1, and Incorrect answers was counted as 0, a mark of ten was calculated

This questionnaire was prepared for the purpose of scientific research under the auspices of the Directorate of Education in Homs governorate..... We thank you for your cooperation

---

Parents questionnaire module was filled by children legal guardians in the purpose of socioeconomic assessment, children health assessment, Healthy and Dietary habits assessment.
