# Supplementary material for: The impact of crisis and COVID-19 on Syrian children growth, health awareness and nutritional practices. a cross-sectional study
Source: BMC Pediatr. 2023 Jun 15;23:295. doi: 10.1186/s12887-023-04115-w (PMC10268436; doi:10.1186/s12887-023-04115-w)
Supplement: Supplementary file 2 — Additional file 2: Table S2. Child questionnaire module. [file 12887_2023_4115_MOESM2_ESM.pdf]

Table S2: Child questionnaire module

Knowledge Score: correct answer was counted as 1, and Incorrect answers was counted as 0, a mark of 10 was calculated

1. food you eat today affects the growth of your body in the future	√	×
2. It is not necessary to have a breakfast before school	√	×
3. vegetables and fruits are important parts of a healthy diet	√	×
4. Meat is a good source of protein	√	×
5. healthy food does not contain vitamins	√	×
6. One of the important ways to protect yourself against Corona disease is Social distance	√	×
7. food pyramid's largest group contains eggs and sugar	√	×

8. Which of the following foods are harmful to dental health?

Milk	Egg	Sugar	Fruit	Fish

9. Which of these foods should be eaten in the least amount?

Bread & rice	Dairy food	Sugar & fat	Vegetables & Fruit	meat

10. Which of the following foods is high in salt and is not healthy?

Chips	Chocolate	Risen	Grapes	All the previous is false

Attitude Score

correct answer was counted as 1, and Incorrect answers was counted as 0, a mark of 8 was calculated

1. Which of the following is a good and healthy option?

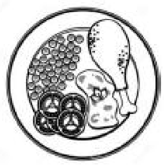
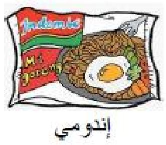

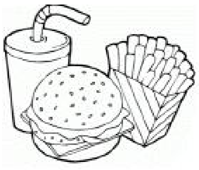
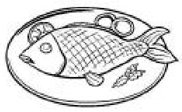
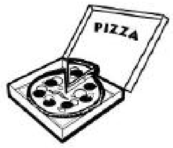

2. Which of the following activities is a good sport for your body?

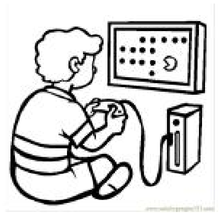
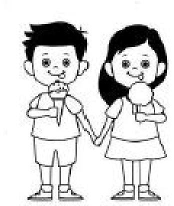
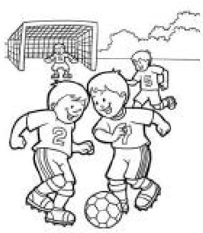
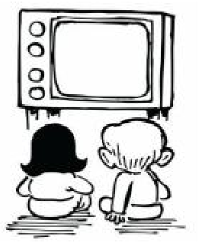

3. If I feel thirsty, which of the following drinks should I drink more?

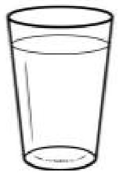
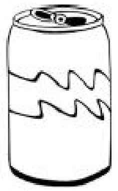
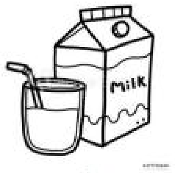

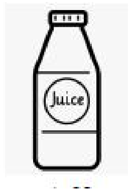

4. Which of the following is a healthy snack?

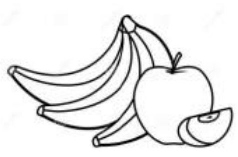
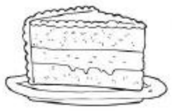
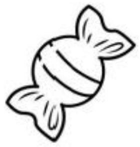
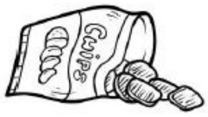

5. Which of the following is unhealthy?

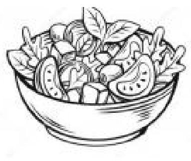
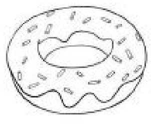
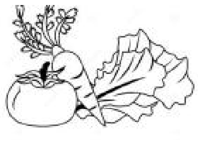
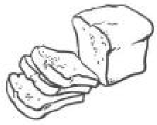
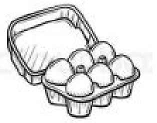

6. Do you prefer to have a breakfast before school?	Yes	No
7.Do you think healthy diet is important for your growth?	Yes	No
8.Do you think it is more healthy to eat Homemade or Store-bought Food	Homemade	Store-bought

How often do you eat vegetables?

several times a day	Once a day	Several times in the week	Once a week	Never eat

How often do you eat fruit?

several times a day	Once a day	Several times a week	Once a week	Never eat

How often do you eat dairy foods?

several times a day	Once a day	Several times a week	Once a week	Never eat

How often do you watch TV?

Every day	Several times a week	Only on holiday	Never watch TV

How often do you do sports?

Every day	Several times a week	once a week	Several times a month	Never

---

Child questionnaire module was filled by third grade students in the purpose of health knowledge assessment, health attitude assessment, and dietary habits assessment.
